# Simultaneous determination of dehydroacetic acid, benzoic acid, sorbic acid, methylparaben and ethylparaben in foods by high-performance liquid chromatography

**DOI:** 10.1007/s10068-023-01264-7

**Published:** 2023-03-20

**Authors:** Ji Sun So, Soo Bin Lee, Jin Hye Lee, Hye Seon Nam, Jong Kwon Lee

**Affiliations:** Food Additives and Packaging Division, National Institute of Food and Drug Safety Evaluation, Cheongju, 28159 Republic of Korea

**Keywords:** Dehydroacetic acid, Benzoic acid, Sorbic acid, Methylparaben, Ethylparaben

## Abstract

In this study, an analytical method was established and validated to determine the preservatives such as dehydroacetic acid, benzoic acid, sorbic acid, methylparaben and ethylparaben. The level of preservatives was measured by solvent extraction method adding purification process with carrez reagent and by high-performance liquid chromatography (HPLC). The developed analytical method was successfully applied to determine the concentration of preservatives in various food samples including jam, cheese and soy sauce, displaying high accuracy (recoveries between 87.8% and 110%) and precision (%RSD less than 5.92% and 7.72% for intra-day and inter-day, respectively). To verify the applicability of the improved test method, selected 13 food items and collected 521 samples were monitored. As a result, all the cases met the Korea standard guidelines. Consequently, this study is expected to contribute to the safety management of preservatives for domestic distribution and imported food.

## Introduction

Preservatives refer to the food additives for preventing a food spoilage resulting from the growth of microorganisms. In Korea, the common preservatives used for food preservation include dehydroacetic acid, sorbic acid, benzoic acid, paraoxybenzoic acid esters, propionic acid and salts thereof (MFDS, 2020). Internationally, the regulations for preservatives are precisely set in terms of the using conditions, the types of foods that can be used and the maximum used amount (Guarino et al., 2011). Each country has different tendency in the commercial preservatives. Further, what can be legally utilized may not be imported, distributed, or sold, since the preservatives do not meet the standards of the domestic food code (Shin, 2016). Therefore, to manage through the accurate preservative inspection process is crucial. In order to comply with forthcoming regulations and to have effective control over food safety, a rapid and reliable method of determining food preservatives is required. Several studies have been undergone on the determination of sorbic acid and benzoic acid in food samples. Many analytical methods, including high performance liquid chromatography (HPLC) (Phechkrajang and Yooyong, 2017; Jang et al., 2018; Timofeeva et al. 2019) and gas chromatography (GC) (Park et al., 2016; Ding et al., 2018; Tungkijanansin et al., 2020) were applied for the determination of these preservatives in various foods. As new sorts of foods continue to emerge and existing foods are tended to be combined with lots of preservatives, a superior purification process which can effectively reduce the food matrix is required. Further, an accurate separation and quantification analyses are needed. Throughout analyzing food, the complex food matrix hinders a quantitative analysis of separation technology. In real samples, the elimination of interfering substances from the food matrix is to be considered significant for accurate measurement of analytes. A sample preparation is applied using several extraction methods, such as liquid–liquid extraction (LLE) (Tighrine et al., 2019), dispersive liquid–liquid microextraction (DLLME) (Sun et al., 2014; Javanmardi et al., 2015; Ding et al., 2018), solid-phase microextraction (SPME) (Tungkijanansin et al., 2020) and solid-phase extraction (SPE) (Win et al., 2019). In the International Dairy Federation (IDF), extraction using only solvent may not sufficiently remove interfering substances, emulsified sample was analyzed using carrez solution (ISO, 2008). In order to efficiently analyze processed foods, among the Spe methods, HLB and C18 cartridges, which are excellent for removing proteins, salts and organic acids, and carrez solution, which are excellent for removing proteins and lipids.

The main objective of this study is to develop and validate an analytical method applicable to all food types where preservatives added (dehydroacetic acid, benzoic acid, sorbic acid, methylparaben, ethylparaben) for safety reasons. Currently, Korea Food Code proposed a steam distillation method and a solvent extraction method. Although the steam distillation is widely applied, it is time-consuming and complicating. Additionally, the solvent extraction method is limited to certain food types, and it does not go through any purification process, which makes the analysis difficult due to the interfering peaks of the matrix. In this study, three purification procedures (SPE(C18, HLB cartridge), carrez reagent solution) were compared in order to establish an optimal simultaneous analysis with five preservatives using HPLC. Further, a monitoring was conducted through the commercially processed foods to verify the applicability with the established analysis method for the preservatives. As a result, using HPLC, this study developed the fastest and simplest simultaneous analysis method for preservatives. The developed analytic method would enable the simultaneous analysis of preservatives (dehydroacetic acid, benzoic acid, sorbic acid, methylparaben, ethylparaben), and could be utilized for all types of food. As the pretreatment process is simple, and the purification effect is excellent, it will save much time and cost.

## Materials and methods

### Sample collection

A total of 521 processed foods were purchased from market, department and online retailers in Republic of Korea. The samples were categorized into 13 categories according to the Korea Food Code: Breads (*n* = 20), sugar processed products (*n* = 20), jams (*n* = 20), edible oils and fats (*n* = 20), beverages (*n* = 67), soy sauces and pastes (*n* = 72), seasoning foods (*n* = 109), pickled foods and stewed foods (*n* = 41), alcoholic beverages (*n* = 20), processed meat products (*n* = 20), milk products (*n* = 45), processed fishery foods (*n* = 55), processed agricultural foods (*n* = 12). The products with preservatives and those without labeling were randomly collected. All samples were homogenised using a blender (Blixer 5 plus, Robot Coupe, Vincennes, France) and stored at –20 °C until analysis.

Meanwhile, validation was conducted by selecting the representative processed foods from food groups with standards for use of the preservatives. Jam was selected as a representative with high in sugar, soy sauce as the one with high salt, and cheese as the one having protein and fat.

### Reagents

The standard-grade dehydroacetic acid, sorbic acid, benzoic acid, methylparaben, ethylparaben, tetra-n-butylammonium hydroxide and phosphoric acid were purchased from Sigma-Aldrich (St. Louis, MO, USA). Ethanol and acetonitrile for an extraction solvent, were obtained from Merck (Frankfurt, Germany). Ultrapure water (resistivity ≥ 18 MΩ) was obtained from a Milli-Q ultrapure water purification system (Millipore, Billerica, MA, USA). Potassium ferricyanide (Sigma-Aldrich, St. Louis, MO, USA) and zinc sulfate heptahydrate (Merck, Frankfurt, Germany) were used as the carrez solution I, carrez solution II. All regents used were analytical grade or better.

### Preparation of solutions

Individual standard stock solutions were prepared by dissolving 100 mg of dehydroacetic acid (DHA), sorbic acid (SA), benzoic acid (BA), methylparaben (MP), ethylparaben (EP) in 100 ml of methanol using 100 ml volumetric flasks. With different concentrations, working standard solutions for DHA, SA, BA at concentrations of 0.3, 0.6, 1.2, 3, 6, 15, 30 and 60 mg/kg and parabens (EP, MP) at concentrations of 0.5, 1, 5, 10, 25, 50 and 100 mg/kg were produced by diluting known aliquots of the stock solutions with methanol. The stock solutions were prepared monthly while the working standard solutions were prepared daily. Carrez solution I was used by dissolving potassium ferrocyanide in water to a concentration of 15%, and Carrez solution II was used by dissolving zinc sulfate in water to a concentration of 30%.

### Comparison of recovery according to purification process

#### SPE method using C18 cartridge

C18 cartridges are known to be excellent at removing salts and organic acids, and have been studied using preservatives (Yun et al., 2019). For the recovery rate test, 40 mg/kg was added to the representative samples (jam, soy sauce, cheese). The solvent extraction of preservatives (DHA, SA, BA, MP, EP) in the samples of jam, soy sauce, cheese followed by respective purification by SPE was performed according to a procedure reported by Yun et al. (2019) with some modifications. In the solvent extraction process, 4 g of the homogenized sample was placed in a conical tube containing 40 ml ethanol and then sonicated for 10 min followed by centrifugation for 10 min at 1500 × g. During the purification, 5 ml of the supernatant was passed through an activated C18 SPE column (Waters, MA, US; the column was preconditioned with 5 ml methanol, 5 ml water and 1 ml acetonitrile before activation) followed by 5 ml of methanol. The total solution was dried using nitrogen, and the analytes were eluted with 1 ml of acetonitrile and filtered through a polyvinylidene fluoride (PVDF) 0.22 μm membrane syringe filter (Teknokroma, Barcelona, Spain).

#### SPE method using HLB cartridge

HLB cartridges are specialized for protein, salt, and lipid removal and comparative experiments were conducted with some modifications referring to the experimental method of Wang et al. (2013). This procedure is identical as the one using C18, and was experimented with only different cartridge by HLB.

### Purification using carrez reagent

The solvent extraction and purification of preservatives in foods by carrez solutions were performed according to the procedure reported by Abedi et al. (2014) with some modifications. During the solvent extraction process, 4 g of the sample was placed in a conical tube. After adding 10 ml of ethanol, 1 ml potassium hexaferrocyanide (Carrez solution I) and 1 ml zinc acetate (Carrez solution II) were added. Then, the closed tube was shaken gently for 1 min to promote formation of a cloudy solution. The resultant was combined with ethanol 40 ml and sonicated for 5 min followed by centrifugation for 10 min at 1500 × g. The supernatant was filtered through PVDF 0.22 μm membrane syringe filter.

### Apparatus and chromatographic conditions

A quantitative analysis was carried out by using HPLC Agilent Technologies 1260 series (Agilent Technologies, CA, USA) equipped with a diode-array detector (DAD). The chromatographic separations were accomplished by using a Capcell pak MF-C8 column (4.6 mm I.d. × 150 mm, 5 μm, Phenomenex, CA, USA). Analytes separation performed with gradient mobile phase composed of 0.01 mol/l tetra-*n*-butylammonium hydroxide, 0.1% phosphoric acid in water (v/v) as eluent A, and acetonitrile as eluent B. The separation with optimized gradient conditions were as follows: Initial, 25% B; 2.5 min, 25% B; 7 min, 35% B; 12 min, 40% B; 12.1 min, 90% B; 15 min, 90% B. The column temperature was maintained at 40 °C, flow rate was 1 ml/min and the injection volume was 10 μl. For the confirmation of five components, LC–MS/MS analysis was performed on the standard and samples under multiple reaction monitoring (MRM) in the negative-ion electro spray ionization (ESI(-)) mode. AB sciex Triple Quad 4500 (Sciex, Framingham, USA) mass spectrometer was combined with Nano space SI-2 3004 (Shiseido, Tokyo, Japan) LC system). The LC–MS/MS settings were as follows: UK025T (2.0 × 150 mm, 3 μm); mobile phase composed of 10 mM ammonium acetate in water (A) and acetonitrile (B); the gradient was initial, 15% B; 13.5 min, 60% B; 14.0 min, 80% B; 16.0 min, 80% B; 16.1 min, 15% B; 20 min, 15% B. The injection volume was 2 μm. DHA, SA, BA, MP and EP were detected at m/z 167/82.9, 121/77, 111/67, 151/91.9, 165/92.3 of precursor/product ions, respectively.

### Method validation

The analytical method for the determination of DHA, SA, BA, MP and EP in the products were validated in terms of the linearity, limit of detection (LOD), limit of quantification (LOQ), selectivity, accuracy (recovery), precision (as percent relative standard deviation (%RSD)) and measurement uncertainty. The method was assessed according to the AOAC (Association of Official Analytical Chemistry) guideline for selectivity, accuracy, precision, LOD, LOQ (AOAC, 2012b), the GUM (guide to the expression of uncertainty in measurement) and EURACHEM guide for the measurement of uncertainty (ISO, 1993; EURACHEM, 2000). Linearity was evaluated using three replicates of calibration curve and calculating the correlation coefficient (r^2^). The LOD and LOQ values were calculated from the calibration curves at eight concentrations. These were determined as 3.3 and 10 σ/S, respectively, where σ refers to the standard deviation of the intercept and S refers to the slope of the regression line determined from the calibration curve. A selectivity was verified by analyzing the chromatograms of standard solution and the three representative samples (jam, cheese, soy sauce) spiked with the stock solution to examine matrix interferences. The accuracy and precision were evaluated by analyzing the three representative samples (jam, cheese, soy sauce) spiked with the stock solution to obtain the final concentrations of 20, 40 and 100 mg/kg. The intra-day (three repetitions on the same day) and inter-day (three repetitions over three different days) precision tests were performed. The measurement uncertainty of the concentration of DHA, SA, BA, MP, EP in processed foods was estimated according to the GUM and EURACHEM guide (ISO, 1993; EURACHEM, 2000). The uncertainty factors were measured based on the four components: The calibration curve, foodstuffs matrix (repeatability), preparation of standards and sample preparation. The concentration of preservatives, respectively at 40 mg/kg, was selected for calculating the uncertainty. The sample repeatability was determined by analyzing jam for 6 times. The measurement uncertainty (*U*), the expanded uncertainty, was calculated by multiplying the combined standard uncertainty by a coverage factor (*k* = 2) that yields a confidence level of approximately 95%.

### Inter laboratory comparison: International proficiency test

To demonstrate the applicability of this study, the international comparative proficiency evaluations were conducted by participating in the Food Analysis Performance Assessment Scheme (FAPAS, Fera Science Ltd., York, UK), a proficiency program operated by the Department for Environment, Food and Rural Affairs in UK. FAPAS analyses the Z score (range of error between laboratories) as an international proficiency test for the 10 fields such as food nutrients, food additives, and pesticide residues, and presents the results. The outcome was evaluated as Z-score, and it was statistically satisfactory if $z\left| \le \right.\,2\left( {z = {{x - x_{a} } \mathord{\left/ {\vphantom {{x - x_{a} } {{\upsigma }_{p} }}} \right. \kern-0pt} {{\upsigma }_{p} }}} \right)$, where x is the participant’s reported result, x_a_ is the assigned value and σ_P_ is the standard deviation for proficiency.

## Results and discussion

### Optimization of HPLC conditions

**Fig. 1:**
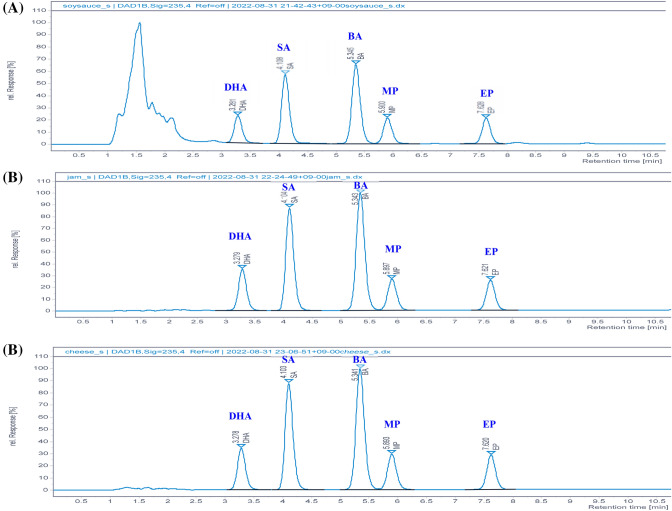
The chromatographic separation of preservatives by wavelength at 235 nm **A** Chromatogram of soysauce spiked with mixed standard solution (40 mg/kg); Retention Time (RT): DHA, 3.043; SA, 4.077; BA, 5.554; MP, 6.046; EP, 7.612. **B** Chromatogram of cheese spiked with mixed standard solution (40 mg/kg); Retention Time (RT): DHA, 3.043; SA, 4.077; BA, 5.554; MP, 6.046; EP, 7.612. **C** Chromatogram of jam spiked with mixed standard solution (40 mg/kg); Retention Time (RT): DHA, 3.043; SA, 4.077; BA, 5.554; MP, 6.046; EP, 7.612

UV scan (200–400 nm) was applied to the analyte in order to minimize the interfering compounds and to evaluate the selectivity of the method. Regarding the detection wavelength, there was a difference in the maximum absorption of the analyte. Korea's Food Code recommended λ = 217 nm for five kinds of preservatives. Nonethless, as a result of the wavelength test, a single wavelength of λ = 235 nm was found to be the best compromise for detecting five preservatives. Compared to 217 nm, the sensitivity of the parabens (Methylparaben, Ethylparaben) decreased slightly at 235 nm, but the sensitivity of sorbic acid was significantly improved (data not shown). A UV scan of the analyte displayed no noticeable differences between the standard solution and the UV full scan (200–400 nm) of the preservative peaks in the processed samples. Five preservatives were well separated with the clear peak by chromatographic conditions. Therefore, this method is proven to have a high selectivity when the actual matrix is applied (Fig. 1).

### Established method

Above all, for the determination of the extraction solvent, the recovery rate test was conducted with four extraction solvents (acetonitrile, acetone, ethanol, methanol) and the resultants were compared. As a result of the solvent test, there was a no significant difference between the solvent recovery rate. However, though a small difference, ethanol showed superiority, and it was selected as the extraction solvent.

As a consequence of comparing the purification process in the three representative foods (jam, cheese, soy sauce), the carrez reagent method generally showed better results in terms of recovery rate and selectivity (Table 1). Although the solid-phase extraction method seemed to be good at removing the processed food matrix, but it seems that there was a loss in recovery during the process of pass through the cartridge and nitrogen concentration step. It is also considered that large variations occurred in these experimental steps. Also SPE cartridge (C18, HLB) approach took longer time due to the time-consuming loading using the pump and nitrogen concentration. Meanwhile the process with carrez reagent had high recovery rates, superior accuracy, variation and it took much less time. Thus, using carrez reagent method was determined as suitable for the purification process. In the case of the ethanol extraction method, since the centrifuged supernatant is analyzed after simple extraction, fat and protein are not sufficiently removed, and in particular, the emulsification properties of emulsified foods such as cheese and butter cannot be destroyed, so the recovery rate is low.

**Table 1:** Comparison of purification process in the representative food

Method	Sample	Recovery (%)					RSD (%)				
		DHA	SA	BA	MP	EP	DHA	SA	BA	MP	EP
Solvent direct extraction	Jam	83.6	85.0	80.5	87.1	77.2	1.2	1.1	1.1	1.2	0.8
	Soysauce	74.8	84.4	77.9	83.5	76.1	1.2	1.6	1.4	1.4	1.6
	Cheese	82.0	85.6	78.0	89.1	78.8	2.2	2.1	2.3	2.0	2.0
SPE C18 catridge	Jam	71.5	69.8	68.9	70.3	70.0	5.4	5.8	6.1	6.6	6.7
	Soysauce	60.1	61.9	63.4	61.8	64.9	6.8	7.0	7.4	7.0	8.1
	Cheese	82.0	79.8	77.6	83.2	82.2	3.9	3.8	3.8	3.4	3.6
SPE HLB catridge	Jam	72.2	70.8	70.7	70.0	71.1	8.0	7.9	8.0	8.0	6.0
	Soysauce	68.6	69.6	71.3	70.1	74.5	4.1	4.0	4.5	4.1	3.9
	Cheese	77.2	74.4	71.5	77.7	76.8	9.9	9.3	10.1	9.3	9.3
Carrez reagent	Jam	100	101	101	101	101	0.9	0.7	0.8	0.8	1.2
	Soysauce	97.7	102	101	99.0	103	0.6	0.3	0.3	0.3	0.3
	Cheese	101	104	102	107	107	2.0	1.7	2.0	1.9	1.6

Carrez solutions are widely used in the purification of proteins and fats, breaking up emulsions that can interfere with the crystallization of small molecules such as carbohydrates (Chávez-Serví et al., 2004; Manzi and Pizzoferrato, 2013; Marconi et al., 2004; Pereda et al., 2009). Therefore, carrez clarification process is optimal for protein precipitation and turbidity removal (Oliveira-Neves et al., 2018). Consequently, the purification process using carrez reagent is considered to be good for various types of food analysis.

### Method validation

The analytical methods showed satisfactory linearity, LOD, LOQ, accuracy and precision. The calibration curves were found to be linear for analytes. The correlation coefficients (*r*^*2*^) of the preservatives displayed over 0.9998. The LODs and LOQs for preservatives are shown in Table 2. The accuracy (recovery) and precision of the method were evaluated using spiked blank samples of jam, cheese, soy sauce at three concentrations, 20, 40 and 100 mg/kg, respectively. The validation results are summarized in Table 2. The recovery of the preservatives is within the range of 87.7–110% for each sample. The intra-day and inter-day precisions are expressed as RSDs and the precisions are in the range of 0.07–7.72%. These recoveries and precisions are within the criteria established by the AOAC guideline (AOAC, 2012b).

**Table 2:** Validation data of the analytical method from the selected samples fortified at 20, 40 and 100 mg/kg with preservatives (DHA, SA, BA, MP, EP) standards

Preservative	Linearity (r^2^)	LOD (mg/kg)	LOQ (mg/kg)	Recovery ± SD (%)		Precision (%RSD)	
				Commodity	Range	Intra-day	Inter-day
DHA	0.9999	0.16	0.49	Soysauce	102 ± 2.12–107 ± 1.95	1.47–3.16	1.02–1.82
				Jam	101 ± 4.04–106 ± 2.91	1.54–4.67	1.59–4.01
				Cheese	104 ± 1.55–106 ± 4.05	0.72–1.48	0.54–3.83
BA	0.9999	0.16	0.48	Soysauce	104 ± 0.64–109 ± 0.69	1.17–2.96	0.61–1.93
				Jam	104 ± 0.96–110 ± 1.75	0.07–3.45	0.25–1.58
				Cheese	92.7 ± 1.66–104 ± 1.21	1.79–4.57	1.16–2.17
SA	0.9999	0.15	0.45	Soysauce	101 ± 2.60–107 ± 1.95	1.02–1.82	1.45–2.97
				Jam	101 ± 4.04–110 ± 2.32	1.59–4.01	1.28–4.96
				Cheese	105 ± 0.31–106 ± 4.05	0.54–3.83	0.29–3.78
MP	0.9999	0.27	0.81	Soysauce	104 ± 0.64–109 ± 0.69	0.61–1.93	1.43–3.19
				Jam	105 ± 0.24–110 ± 1.75	0.25–1.58	0.23–3.58
				Cheese	97.3 ± 1.98–104 ± 1.21	1.16–2.17	2.04–4.39
EP	0.9998	0.31	0.93	Soysauce	93.3 ± 5.52–101 ± 3.30	3.27–5.92	1.00–7.72
				Jam	87.7 ± 2.98–104 ± 5.43	0.84–5.57	3.40–5.81
				Cheese	90.7 ± 3.39–99.4 ± 7.09	2.12–4.47	3.74–7.14

SD^*^ standard deviation

The measurement uncertainty of the concentration of analytes in the processed foods, expressed as mg/kg, was calculated from the expanded standard uncertainty, with a coverage factor of *k* = 2, which indicates approximate 95% confidence. The total relative uncertainty was expressed based on the combined relative uncertainty of four factors: Calibration curve, sample matrix (repeatability), preparation of standards and sample preparation. The standard uncertainty was calculated by combining the relative uncertainty and the concentration of the analyzed jam (40 mg/kg). The expanded uncertainty was calculated based on the combined standard uncertainty. The final analyzed concentrations of DA, SA, BA, MP, EP in jam were 44.7 ± 2.2, 44.0 ± 2.1, 44.4 ± 2.0, 44.3 ± 2.1 and 43.4 ± 2.2 mg/kg, respectively. The results for each factor of measurement uncertainty are summarized in a Table 3.

**Table 3:** Measurement uncertainty calculation result of preservatives (DHA, SA, BA, MP, EP)

	DHA	SA	BA	MP	EP
Effective degree of freedom (V_eff_)	44.7	44	44.4	44.3	43.4
Total relative uncertainty (U_r_/r)	0.02	0.02	0.02	0.02	0.03
Combined standard uncertainty (mg L^−1^) (U_c_)	1.1	1.1	1	1.1	1.1
Expanded uncertainty (mg L^−1^) (U)	2.2	2.1	2	2.2	2.2
*k* (confidence interval approximately 95%)	2	2	2	2	2
Final analysis result (mg L^−1^)	44.7 ± 2.2	44.0 ± 2.1	44.4 ± 2.0	44.3 ± 2.1	43.4 ± 2.2

### International proficiency test results

The analyzed sample for the first international comparative proficiency evaluation was selected as soft drink (soft drink, 03160) and the z-score was 0.5 for sorbic acid and −0.6 for benzoic acid. The second analysis sample was jam (Jam, 20171), which was evaluated as −0.2 sorbic acid and 0.0 benzoic acid. The z-score (range of error between laboratories) ≤ 2 was considered statistically satisfactory. These results were to confirm the accuracy of the experimental method used in this study.

### Monitoring results of preservatives in domestic processed foods

**Fig. 2:**
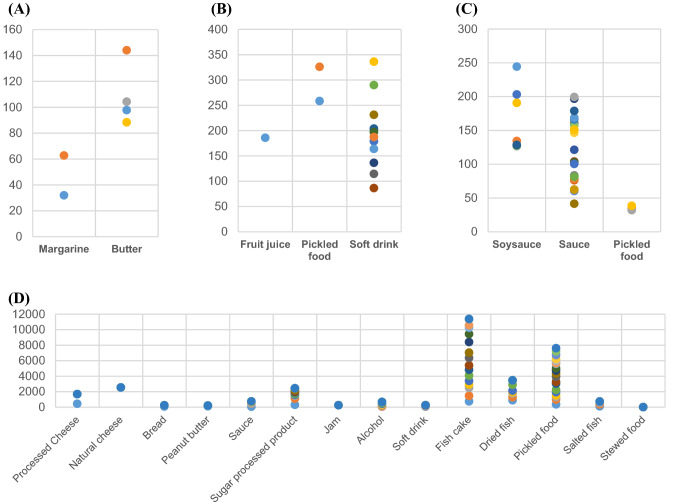
Distribution of detection of preservatives. **A** Distribution of detecting dehydroacetic acid. **B** Distribution of detecting benzoic acid. **C** The detection distribution of ethylparaben showed a wide range of 41.5–244 mg/kg. **D** As for the detection distribution of sorbic acid, it showed a wide range from 312 to 1,363 mg/kg for the processed fish products and 86.6 to 791 for the pickled food products

Table 4 represents the result of monitoring to verify the applicability of the established method with carrez reagent. Figure 2 shows the distribution of each component for the monitoring results. Among the collected products, 2 out of 10 butter and 6 out of 10 margarine products were found to be combined with DA. DA was detected in all the six cases of the labeling, and the range was 32–144 mg/kg, meeting the regulations of 500 mg/kg (Korean standard).

**Table 4:** Concentration of preservative in food samples (mg/kg) analysed by HPLC–DAD

Commodity	Tested Sample	Dehydroacetic acid			Benzoic acid			Sorbic acid			Ethyl p-hydroxybenzoate		
		Detected sample	Range (mg/kg)	Detected mean ± SD (mg/kg)	Detected sample	Range (mg/kg)	Detected mean ± SD (mg/kg)	Detected sample	Range (mg/kg)	Detected mean ± SD (mg/kg)	Detected sample	Range (mg/kg)	Detected mean ± SD (mg/kg)
Processed Cheese	15	ND	ND	ND	ND	ND	ND	2	ND-1238	837 ± 401	ND	ND	ND
Soy sauce	22	ND	ND	ND	ND	ND	ND	ND	ND	ND	7	ND-244	165 ± 44.0
Dried fish	15	ND	ND	ND	ND	ND	ND	7	ND-876	494 ± 244	ND	ND	ND
Dried and stored meat	10	ND	ND	ND	ND	ND	ND	ND	ND	ND	ND	ND	ND
Red pepper paste	15	ND	ND	ND	ND	ND	ND	ND	ND	ND	ND	ND	ND
Fruit juice	15	ND	ND	ND	1	ND-186	186 ± 0	ND	ND	ND	ND	ND	ND
Sugar processed products	20	ND	ND	ND	ND	ND	ND	11	ND-801	221 ± 208	ND	ND	ND
Soybean paste	15	ND	ND	ND	ND	ND	ND	ND	ND	ND	ND	ND	ND
Peanut butter	12	ND	ND	ND	ND	ND	ND	2	ND-107	98.2 ± 9.30	ND	ND	ND
Margarine	10	2	ND-62.7	47.3 ± 15.4	ND	ND	ND	ND	ND	ND	ND	ND	ND
Mayonnaise	12	ND	ND	ND	ND	ND	ND	ND	ND	ND	ND	ND	ND
Imitation Cheese	10	ND	ND	ND	ND	ND	ND	ND	ND	ND	ND	ND	ND
Butter	15	4	ND-144	108 ± 21.2	ND	ND	ND	ND	ND	ND	ND	ND	ND
Bacon	10	ND	ND	ND	ND	ND	ND	ND	ND	ND	ND	ND	ND
Bread	20	ND	ND	ND	ND	ND	ND	2	ND-155	116 ± 38.6	ND	ND	ND
Sauce	36	ND	ND	ND	ND	ND	ND	5	ND-402	146 ± 142	23	ND-200	116 ± 47.1
Vinegar	20	ND	ND	ND	ND	ND	ND	ND	ND	ND	ND	ND	ND
Fish cake	20	ND	ND	ND	ND	ND	ND	15	ND-1363	757 ± 260	ND	ND	ND
Natural cheese	15	ND	ND	ND	ND	ND	ND	1	ND-2532	2532 ± 0	ND	ND	ND
Jam	20	ND	ND	ND	ND	ND	ND	1	ND-256	252 ± 0	ND	ND	ND
Pickles	26	ND	ND	ND	2	ND-326	292 ± 33.9	19	ND-791	400 ± 169	3	ND-38.4	35.2 ± 2.61
Salted fish	20	ND	ND	ND	ND	ND	ND	4	ND-260	180 ± 61.0	ND	ND	ND
Stewed food products	15	ND	ND	ND	ND	ND	ND	1	ND-7.00	7.00 ± 0	ND	ND	ND
Alcohol	20	ND	ND	ND	ND	ND	ND	7	ND-123	95.9 ± 37.2	ND	ND	ND
Soft drink	22	ND	ND	ND	12	ND-336	194 ± 66.7	4	ND-71.9	63.3 ± 7.36	ND	ND	ND
Tomato ketchup	20	ND	ND	ND	ND	ND	ND	ND	ND	ND	ND	ND	ND
Spice products	21	ND	ND	ND	ND	ND	ND	ND	ND	ND	ND	ND	ND
Mixed drink	20	ND	ND	ND	ND	ND	ND	ND	ND	ND	ND	ND	ND
Mixed paste	20	ND	ND	ND	ND	ND	ND	ND	ND	ND	ND	ND	ND
Red ginseng drink	10	ND	ND	ND	ND	ND	ND	ND	ND	ND	ND	ND	ND
Total	521	4	ND-144	47.3 ± 21.2	15	ND-336	186 ± 66.7	81	ND-1,238	7.00 ± 401	33	ND-244	35.2 ± 47.1

*SD* standard deviation; *ND* not detected

As SA is the most widely used preservative, it is used in various ways, and it was found in diverse items such as cheese, fish cake, dried fish, pickles, sauces, syrup, jam, bread, and alcoholic beverages. A total of 114 items with SA on the labeling were collected: Processed cheese 10 cases, natural cheese 1 case, dried fish paste 8 cases, jam 1 case, sugar processed products 11 cases, peanut butter 2 cases, bread 3 cases, sauces 10 cases, fish meat products 15 cases, pickled food 19 cases, stewed food 1 case, salted fish 4 cases, alcoholic beverages 16 cases and carbonated drinks 4 cases. Among the labeled products, eight pickled foods, one dried fish, five sauces, one bread and nine alcoholic beverages were not detected as the components were less than the LOQ. SA showed the lowest detected amount of 7 mg/kg in stewed foods, and the highest detected amount of 2532 mg/kg in natural cheese. All of them were suitable for regulations for use, except bread as it was included in the product group without standard for use. Nonethless, due to margarine, the raw material, and it was judged to be suitable (the use standard did not exceed 2000 mg/kg).

As the product labeled with BA, 1 fruit and vegetable juice, 3 stewed foods, 4 pickled foods, and 12 carbonated beverages were collected. BA was found to be particularly widely used in the beverages. Among the labeled products, 3 stewed foods and 1 pickled food were classified as not detected, as they were below LOQ. The standard for use of BA was 600 mg/kg in fruit and vegetable juice and soft drink, and 1000 mg/kg in pickled foods.

Among the processed foods in the market, the products containing MP were not collected and detected in all foods.

As the product labeled with EP, a total of 40 items were collected: 7 soy sauce, 26 sauces, 5 pickled foods, and 2 stewed foods. It was found that they are relatively widely used in the sauces. The standard for use of EP is 250 mg/kg in soy sauce and 200 mg/kg in sauces, pickled foods, and stewed foods. Except for the seven cases detecting the value below the LOQ (three cases from sauces, two cases from stewed foods, and two cases of pickled foods), all were suitable for the regulations.

In this study, sorbic acid was detected at 2532 mg/kg in natural cheese, which was within the standard, but at a very high level, followed by 1363.3 mg/kg in fish products, 1238 mg/kg in processed cheese, 801.1 mg/kg in syrup, and 791.3 mg/kg in pickles. According to Lee et al. (2013) previous study, processed cheese was detected the highest at 761.7 mg/kg, processed fish meat products 712 mg/kg, dried stored meat 585.3 mg/kg, salted seafood 492.4 mg/kg, and sausage 369.5 mg/kg. However, there were some differences in other items such as salted fish and sausages. In this study, benzoic acid was detected at 206.5 mg/kg in most beverages except for pickles in two cases. Lee et al. (2013)reported benzoic acid was detected at 86.5 mg/kg in beverages and 13.6 mg/kg in pickles, showing a big difference, but like this study, it was not detected in jams.

According to Shin et al. (2017), the intake risk is evaluated by the estimated daily intake (EDI), and the main causes of EDI for benzoic acid are beverages, and the main causes of EDI for sorbic acid are soybean paste, processed fish products, and pickles. In addition, Yoon et al. (2001) also reported that the exposure of benzoic acid is mostly due to beverages, so regular monitoring of beverages is necessary.

In this study, as a result of randomly collecting and examining processed foods sold in the Korean market, sorbic acid was not detected in all soybean paste, but was detected in 7 out of 25 cases in processed fish products, reaching ND-876 mg/kg and ND-791 mg/kg in 19 cases out of 26 in pickles. It was confirmed that sorbic acid is commonly used in pickles. The detection amount was all within the standard and was at a safe level. Benzoic acid was detected in beverages in 12 out of 22 cases, and the detected amount was ND-336 mg/kg, which was also far below the standard, confirming that it was a safe level. By conducting monitoring using such a quick and accurate method, it can be used as research data for the safe use of food additives through continuous research.

## Conclusions

In this study, an analytical method for the simultaneous determination of preservatives (dehydroacetic acid, benzoic acid, sorbic acid, methylparaben, ethylparaben) in different kinds of foodstuffs by HPLC–DAD was utilized. The chromatography coupled to conductivity detection was developed, validated and applied for the analysis of the commercial samples. The usage of carrez reagent, an excellent material for removing interfering substances, was found to be extremely suitable for this study. The pre-treatment method using carrez reagent showed high recovery, time-consuming, rapid and easy. Currently, the Korea Food Code recommends the steam distillation method and the solvent extraction method limited to livestock products. The stream distillation methods were used widely among the analytical laboratories (AOAC, 2012a), but impossible to process at the same time, so time-consuming and costly in solvents (Techakriengkrai and Surakarnkul, 2007). Analysis of the centrifuged supernatant after just solvent extraction for processed food analysis does not sufficiently remove fat and protein. In particular, there is a problem that the recovery rate is low because it cannot destroy the emulsification properties of emulsified foods such as cheese and butter. Through this study, the test method that expands the range of applicable foods will be revised by adding a purification process of carrez reagent to the solvent extraction method. In addition, this study result will help to improve the preservative test method and to establish a database in the near future. Therefore, building a database through continuous monitoring of raw materials and expanding the scope of application of experimental methods will be great help in providing a rapid and efficient preservative analysis method.
